# Transplantation of neural precursors generated from spinal progenitor cells reduces inflammation in spinal cord injury via NF-κB pathway inhibition

**DOI:** 10.1186/s12974-019-1394-7

**Published:** 2019-01-17

**Authors:** Kristyna Karova, John V. Wainwright, Lucia Machova-Urdzikova, Rishikaysh V. Pisal, Meic Schmidt, Pavla Jendelova, Meena Jhanwar-Uniyal

**Affiliations:** 1 Institute of Experimental Medicine, Czech Academy of Sciences, Videnska 1083, 142 20 Prague, Czech Republic; 2 New York Medical College, Valhalla, NY 10595 USA; 3 2nd Faculty of Medicine, Charles University, V Uvalu 84, 150 06 Prague, Czech Republic

**Keywords:** NF-κB, p65, Spinal cord injury, Stem cells transplantation, Inflammation, TNF-α

## Abstract

**Background:**

Traumatic spinal cord injury (SCI) triggers a chain of events that is accompanied by an inflammatory reaction leading to necrotic cell death at the core of the injury site, which is restricted by astrogliosis and apoptotic cell death in the surrounding areas. Activation of nuclear factor-κB (NF-κB) signaling pathway has been shown to be associated with inflammatory response induced by SCI. Here, we elucidate the pattern of activation of NF-κB in the pathology of SCI in rats and investigate the effect of transplantation of spinal neural precursors (SPC-01) on its activity and related astrogliosis.

**Methods:**

Using a rat compression model of SCI, we transplanted SPC-01 cells or injected saline into the lesion 7 days after SCI induction. Paraffin-embedded sections were used to assess p65 NF-κB nuclear translocation at days 1, 3, 7, 10, 14, and 28 and to determine levels of glial scaring, white and gray matter preservation, and cavity size at day 28 after SCI. Additionally, levels of p65 phosphorylated at Serine536 were determined 10, 14, and 28 days after SCI as well as levels of locally secreted TNF-α.

**Results:**

We determined a bimodal activation pattern of canonical p65 NF-κB signaling pathway in the pathology of SCI with peaks at 3 and 28 days after injury induction. Transplantation of SCI-01 cells resulted in significant downregulation of TNF-α production at 10 and 14 days after SCI and in strong inhibition of p65 NF-κB activity at 28 days after SCI, mainly in the gray matter. Moreover, reduced formation of glial scar was found in SPC-01-transplanted rats along with enhanced gray matter preservation and reduced cavity size.

**Conclusions:**

The results of this study demonstrate strong immunomodulatory properties of SPC-01 cells based on inhibition of a major signaling pathway. Canonical NF-κB pathway activation underlines much of the immune response after SCI including cytokine, chemokine, and apoptosis-related factor production as well as immune cell activation and infiltration. Reduced inflammation may have led to observed tissue sparing. Additionally, such immune response modulation could have impacted astrocyte activation resulting in a reduced glial scar.

**Electronic supplementary material:**

The online version of this article (10.1186/s12974-019-1394-7) contains supplementary material, which is available to authorized users.

## Background

Acute traumatic spinal cord injury (SCI) is a leading cause of disability. Following the primary injury, secondary injury begins, which includes a strong inflammatory response that governs the final outcome of SCI. Such inflammatory response is important for the clearance of cellular debris, which can prevent the regeneration of surviving axons. However, prolonged inflammation can damage healthy tissue and worsen the injury. In response to released cytokines from immune cells, perilesional astrocytes modify their phenotype and form the glial scar composed of astrocytes and a dense extracellular matrix within weeks following SCI [1]. Accordingly, a major hurdle in recovery from SCI is the upregulation of certain inflammatory molecules after an injury that results in gliosis.

Several pathways are involved in immune response after trauma, one of them is the canonical NF-κB (p65) pathway which is particularly implicated in the production of pro-inflammatory cytokines and chemokines by glial cells and infiltrated immune cells [2, 3]. An important role of the NF-kB pathway in the activation of astrocytes and the formation of the glial scar has been suggested [4, 5]. NF-κB pathway is triggered by several stimuli in the central nervous system (CNS), most important of which are Toll-like receptors responding to released danger-associated molecular patterns (DAMPs) [6], pathogen-associated molecular patterns (PAMPs), and members of tumor necrosis factor receptor (TNFR) family, which are present on all cells of the CNS with various specificity. Tumor necrosis factor-α (TNF-α) is produced in injured spinal cords where it binds to TNFR1/TNFR2, to activate the downstream NF-κB signaling pathway [4, 7, 8]. Apart from inflammation, the formation of a glial scar is one of the most prominent host reactions after injury of the CNS including the spinal cord. Astrocytes become activated, proliferate and transform into fibrous elongated cells, which form an intertwined interface between injured and healthy tissue [9]. These are characterized by robust upregulation of glial fibrillary acidic protein (GFAP) [10–13]; its expression is also associated with transcription factors STAT-3 [9, 14], NF-κB [4], and JNK [15]. While it has been shown that the presence of activated astrocytes after SCI is vital for regeneration [16], a dense glial scar that forms after injury remains a barrier that hampers axonal growth across the lesion. Therefore, a balanced solution preventing the scar formation while preserving beneficial properties of astrocytes needs to be developed.

A number of studies have focused on the treatment of SCI with various types of stem cells with varied but promising results [17–20]. Studies have demonstrated that neural stem cells or their conditioned medium modifies the immune response via reduced production of pro-inflammatory cytokines TNFα, IL-1β, and IL-2 and chemokines MIP1α and RANTES [17, 21] while decreasing the production of iNOS [22]. Understanding the underlying mechanisms of the therapeutic effects of these stem cells is of crucial significance, as their modifications can be used as a component of stem cell therapy. In our previous study, we used neural precursors generated from fetal spinal cord (SPC-01) and observed good survival and improvements of motor function [23]. Here, we investigate the impact of SPC-01 on the regulation of inflammatory response, specifically on the modulation of canonical p65 associated NF-κB pathway in the recovery from SCI.

## Methods and materials

### Animals

All experiments were performed in accordance with the European Communities Council Directive of 22nd of September 2010 (2010/63/EU) regarding the use of animals in research and were approved by the Ethics Committee of the Institute of Experimental Medicine ASCR, Prague, Czech Republic. The number of animals was statistically optimized for each particular experiment to achieve its reduction.

Male Wistar rats (*n* = 87) weighing 270–300 g were used in this study. Following SCI, rats were randomly divided into groups surviving 1, 3, 7, 10, 14, or 28 days. On day 7, rats were transplanted with 5 × 10^5^ SPC-01 cells or they were given saline. For immunohistochemical analysis of NF-κB p65 nuclear translocation, gray and white matter sparing, and cavity size, three to six rats per treatment group were used (*n* = 44), along with three uninjured controls for NF-κB p65 analysis; for the assessment of glial scarring 28 days after SCI, four animals per group were used (*n* = 8). For NF-κB activity assay (ELISA), which was determined 10, 14, and 28 days after SCI as well as in uninjured controls, six animals per treatment group were used (*n* = 40) and four uninjured controls. For TNFα analysis, five animals per group and time point were used (*n* = 30).

### Cell cultures

Human spinal cord cell line SPC-01 was produced from a 10-week-old human fetal spinal cord. Obtained cells were conditionally immortalized with a c-mycER^TAM^ construct and were also transduced with GPF using a lentivirus as a vector. A more detailed procedure description can be found here [24]. SPC-01 were grown in culture flasks coated with laminin (10 μg/ml, Sigma, Darmstadt, Germany) and media containing DMEM/F12 (Gibco, Life Technologies, Grand Island, NY, USA), human apo-transferrin (100 μg/ml), human putrescine dihydrochloride (16.2 μg/ml), sodium selenite (40 ng/ml), human recombinant insulin (5 μg/ml), progesterone (60 ng/ml), l-glutamine (2 mM), 4-hydroxy-tamoxifen (100 nM) (each of the foregoing from Sigma, Darmstadt, Germany, unless where otherwise specified above), and human EGF and bFGF (20 ng/ml and 10 ng/ml, respectively) both from Peprotech EC, London, UK. Media was changed every third day, and cells were harvested at 60–70% confluence.

### Spinal cord injury

A balloon-induced compression lesion model was developed to simulate the environmental situation and pathology of spinal cord tissue in the spinal canal, resembling the majority of spinal cord injury causes in humans [25].

To obtain reproducible and standard results, the weight and sex of the animals as well as the size of the balloon and the duration it is inflated must be kept constant among procedures. The ideal weight of male Wistar rats has been established as being in the range of 270–300 g (approx. 10-week-old animals). Animals were anesthetised with an inhalant mixture of isoflurane and air (3% *v*/*v* isoflurane, AbbVie), with an inflow of 300 ml/min, and kept under anesthesia for the entire time of the procedure. After hair removal on the animal’s back, an incision was made at the thoracic level. Carefully, vertebrae (T9, T10, T11) were exposed, and laminectomy of the T10 vertebrae was performed. A 2-French Fogarty catheter was inserted into the epidural space through the created entry site. To produce a T8 lesion, the catheter was inserted 1 cm cranially and the balloon was then inflated with 15 μl of saline for 5 min. Care was taken to ensure that no air bubbles were present in the balloon. After the lesion was complete, the catheter was deflated and removed and the wound was sutured in anatomical layers. Body temperature was continuously monitored with a rectal thermometer and maintained at 37 °C using a heating pad. Rats were given antibiotics (Gentamicin, Lek Pharmaceutical, 5 mg/kg, i.m.) and analgetics (carprofen, Rimadyl, Pfizer, 7.5 mg/kg, i.m.) for 10 days to prevent post-operative infection and to reduce pain. On day 6 (1 day prior to transplantation), immunosuppression regime was started using cyclosporine A (ciclosporin, Novartis, 10 mg/kg i.p.) and azathioprine sodium (Imuran, Aspen, 4 mg/kg, p.o. and 2 mg/kg p.o. after transplantation) to prevent rejection. Animals were checked on daily, and their urine was manually expressed twice daily until this function was recovered, typically 5–8 days. Rats were housed in IVC cages with a 12-h light/dark cycle and had unlimited access to water and food pellets.

### Cell transplantation

Immortalized spinal precursors expressing GFP (SPC-01-GFP) were injected in the lesion site 7 days after spinal cord injury. Animals were anesthetised and fixed in a stereotactic frame (Stoelting Co., Wood Dale, IL, USA), and their spinal cords were carefully exposed at Th8. Using Nano-Injector (Stoelting Co., Wood Dale, IL, USA) and a glass pipette, 5 × 10^5^ cells in 5 μl of saline were transplanted at a rate of 1 μl/min. The glass pipette was kept in place for another 5 min to prevent backward leaking of cell suspension. Control animals received 5 μl of saline.

### Tissue collection

At days 1, 3, 7, 10, 14, or 28 after spinal cord injury (or 3, 7, and 21 days after transplantation), rats were transcardially perfused with 4% paraformaldehyde for 15 min. The spinal columns were removed and kept in 4% paraformaldehyde for 12 h with subsequent dissection of the spinal cords. Dissected spinal tissue was kept in sterile PBS until paraffin embedding.

### Immunohistochemistry

Paraffin-embedded spinal cords were cut into 5-μm-thick cross-sections. A total of 15 sections 1 mm apart were used from each animal amounting to 1.5 cm of tissue used for analysis of NF-κB (p65) nuclear translocation. Time points of 1, 3, 7, 10, 14, or 28 days (*n* = 5 for each group and time point) after SCI were used to visualize the expression of p65, for which primary rabbit anti-p65 IgG antibody (Santa Cruz, Dallas, TX, USA) and secondary goat anti-rabbit IgG antibody conjugated with horseradish peroxidase H (PK-6101, Vector Laboratories, Burlingame, CA, USA) were used. DAB (3′-diaminobenzidine) and H_2_O_2_ were added to produce brown precipitate where secondary antibody was present. The sections were subsequently stained with hematoxylin to visualize cell nuclei. For tissue sparing analysis and cavity size, the same sections were used. For immunohistochemical assessment of glial scarring, the spinal sections (14 cross sections, 5 μm, 1 mm apart, total sample length was 1.5 cm) from animals surviving for 28 days after SCI (*n* = 10) were used for staining with mouse anti-GFAP-Cy3 IgG (C-9205, Sigma-Aldrich, St. Louis, MO, USA).

### Microscopy and image analysis

Microscope LEICA CTR 6500 with software FAXS 4.2.6245.1020 (TissueGnostics, Vienna, Austria) was used to take images of the spinal cross-sections. The density of translocated p65 into the cell nuclei per square millimeter was analyzed by software HistoQuest 4.0.4.0154 (TissueGnostics, Vienna, Austria). Only DAB-positive nuclei were included in the analysis. Area of spared tissue or lesion was obtained automatically using HistoQuest 4.0.4.0154 (TissueGnostics, Vienna, Austria). SPC-01 grafts formed dense clusters and were clearly distinguishable and were excluded from tissue sparing analysis (see Additional file 1). Lesion volume was calculated by multiplying lesion area (mm^2^) obtained using HistoQuest 4.0.4.0154 by the number of lesioned spinal cord sections, which were 1 mm apart.

### Measurement of secretory cytokine concentrations

Levels of the secretory inflammatory cytokine, TNF-α, after saline treatment or transplantation with SPC-01 cells were determined 10, 14, and 28 days after SCI. The spinal cords were removed, and a 2-mm-long segment from the center of the lesion was dissected and incubated in cell culture media (DMEM, Sigma, St. Louis, MO, USA), supplemented with 10% FBS and 0.2% primocin for 24 h; thereafter, secretory cytokine levels were detected in the collected media. A customized Milliplex inflammatory cytokine kit (Millipore, Billerica, MA, USA) was used for magnetic bead detection on the Luminex xMAP instrument and using xPONENT software to analyze the levels of TNF-α. The assays were performed in 96-well filter bottom plates according to the manufacturer’s protocol. Antibody conjugated beads were used at a concentration of 5000 beads per marker and were incubated overnight with samples. A biotinylated detection antibody with streptavidin-R-Phycoerythrin (streptavidin-RPE) (Life Technologies, Carlsbad, CA, USA) was used to measure the levels of TNF-α on a Luminex xMAP 200 system (Luminex, Madison, WI, USA). Data were processed using Magpix instrumentation software. Mean fluorescence intensity (MFI) was used to calculate the concentration of TNF-α; a four- or five-parameter logistic fit curve was generated from seven standards. The lowest standard, at least three times above the background, was used to determine the lower limit of quantification (LLOQ). The LLOQ was calculated by subtracting the MFI of the background (diluent) from the MFI of the lowest standard concentration and back-calculating the concentration from the standard curve.

### ELISA

NF-κB activity was determined by NF-kB p65 (pS536)/Total NF-κB p65 SimpleStep ELISA™ immunoassay kit for the semi-quantitative measurement of NF-kB p65 (pS536) activity (Abcam, Cambridge, MA, USA) according to the manufacturer’s instructions. A 2-mm-long segment from the center of the lesion was homogenized in lysis buffer with phosphatase and protease inhibitors. Protein concentrations were determined using a modified Bradford assay (Bio-Rad, Hercules, CA, USA). The assay was performed by adding samples or standards to the wells, followed by the antibody mix. After incubation, the wells were washed to remove unbound material. TMB substrate was added and catalyzed by horseradish peroxidase, generating a blue dye. This reaction was then stopped by the addition of Stop Solution to develop a color change from blue to yellow. Signal was generated proportionally to the amount of bound analyte, and the absorbance was measured at 450 nm. Values of phosphorylated NF-kB p65 (pS536) over total NF-kB were calculated from the concentrations determined using the standard curve method.

### Statistical analysis

To assess statistically the significant differences in NF-κB activity between treatment groups or location within injured spinal cords as well as in glial scar analysis and TNFα, two-way ANOVA with Bonferroni posttests was used. For ELISA and glial scar statistical analysis, unpaired or paired *t* test was used (GraphPad Prism 5, La Jolla, CA, USA). Differences were considered significant if *p* < 0.05. All data are shown as means ± standard error of the mean.

## Results

### NF-κB activity in the developing spinal cord lesion

**Fig. 1:**
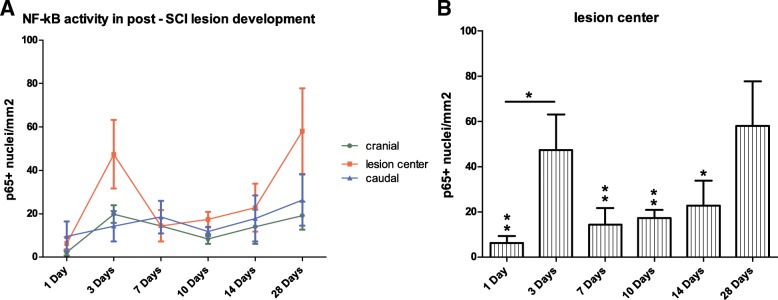
Pattern of NF-κB activity in the lesion center and adjacent cranial and caudal areas at 1, 3, 7, 10, 14, and 28 days after SCI, presented as a number of NF-kB p65^+^ nuclei per square millimeter (**a**). The most pronounced levels of NF-kB p65 nuclear translocation were detected in the lesion centers at day 28 after SCI and were found to be significantly higher than all other time points except for values from day 3 after SCI. In addition, NF-κB-related transcription was significantly higher 3 days after injury when compared with 1-day interval (**b**). Data are shown as the mean and SEM, *p ≤ 0.05, **p ≤ 0.01

Our results of immunohistochemical analysis of the spinal cords at 1, 3, 7, 10, 14, and 28 days after SCI indicate that NF-κB (p65) nuclear translocation is most pronounced in the central areas of spinal cord lesions with mild increase cranially and caudally. NF-κB (p65) nuclear translocation is elevated in the cranial and caudal parts of the injury and remains at similar levels in all time points (Fig. 1a). However, the course of p65 nuclear translocation in the lesion center shows two peaks, first at day 3 after SCI and second at day 28 after injury reaching the highest value (Fig. 1a, b). We observed a strong NF-κB activity at the last time point (day 28), which was significantly higher than all other time points with the exception of values 3 days after induction of SCI, and NF-κB nuclear translocation was detected to be significantly higher at 3 days after SCI as compared to 1 day (Fig. 1b).

### NF-κB nuclear translocation is inhibited after SPC-01 transplantation

**Fig. 2:**
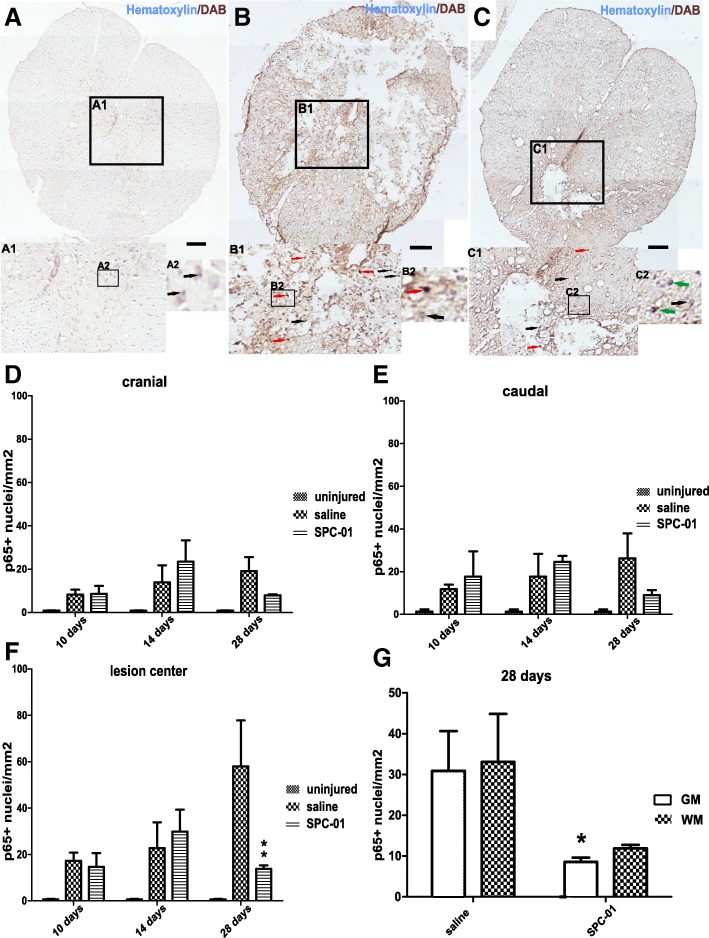
Immunohistochemical staining with hematoxylin and DAB (NF-kB p65) of the spinal cord from uninjured rats (**a**; A1, A2), 28 days after injury from rats treated with saline (**b**; B1, B2), or 28 days from rats transplanted with SPC-01 (**c**; C1, C2). Black arrows point to the nuclei void of NF-kB p65 expression, red arrows highlight the nuclei with translocated p65, and green arrows indicate cells with nuclei negative to p65 surrounded by p65^+^ cytoplasm (A1, A2, B1, B2, C1, C2). NF-κB nuclear translocation in the cranial and caudal areas did not differ significantly between the treatment groups (**d**, **e**). In the lesion center of the spinal cords, a significantly lower NF-κB p65 activity was observed at 28 days in the group treated with SPC-01 cells (**f**). A comparison of nuclear expression of NF-κB in the entire studied areas (gray and white matter) of injured spinal cords at day 28 after SCI showed lower p65 nuclear translocation in SPC-01-treated rats, which was more prominent in the gray matter of the injured spinal cords (**g**). Data are shown as the mean and SEM, *p ≤ 0.05, ** *p* ≤ 0.01. Scale bars, 200 μm

Noticeably, low levels of NF-κB nuclear translocation were detected in the spinal cords of uninjured rats (Fig. 2a). After compression injury, NF-κB (p65) activity increases both in the saline-treated and SPC-01-treated groups at days 10 and 14 after SCI (Fig. 2b, c). However, rats transplanted with SPC-01 cells showed markedly lower NF-kB p65 nuclear translocation than rats receiving saline at 28 days after injury, especially in the gray matter of the spinal cords (Fig. 2c (C1), g). The activity pattern was comparable in areas cranially and caudally to the lesion center 10 and 14 days after SCI with an increase in p65 nuclear translocation. A decrease, which did not reach statistical significance, was observed in the SPC-01-treated group at 28 days post-SCI both cranially and caudally from the central area of the lesion while levels in saline-treated animals remained similar to earlier time points (Fig. 2d, e). In the lesion center, both treatment groups had elevated levels of nuclear translocated p65 NF-kB at days 10 and 14, which continued to rise in saline-treated animals as a contrast to the SPC-01-treated rats, which were reduced to significantly lower levels at 28 days after SCI (Fig. 2f).

### SPC-01 cells reduce levels of p65 NF-κB^pSerine536^

**Fig. 3:**
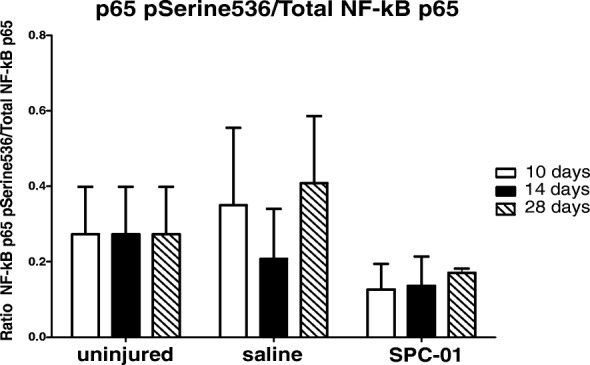
The ratio of phosphorylated NF-κB p65ser536 to total p65 was measured at 10, 14, and 28 days after SCI. No significant change in the activity between the treatment groups was detected. Data are shown as mean and SEM

Levels of nuclear p65 phosphorylated at Serine536 site over total nuclear p65 were measured using an activity assay. The ratio of phosphorylated to total p65 revealed a significantly decreased levels of activated NF-κB p65 in rats treated with SPC-01 cells at all time points. Statistical significance was reached 28 days after SCI when compared with the corresponding saline controls (Fig. 3).

### Locally secreted TNF-α is reduced in SPC-01-treated rats

**Fig. 4:**
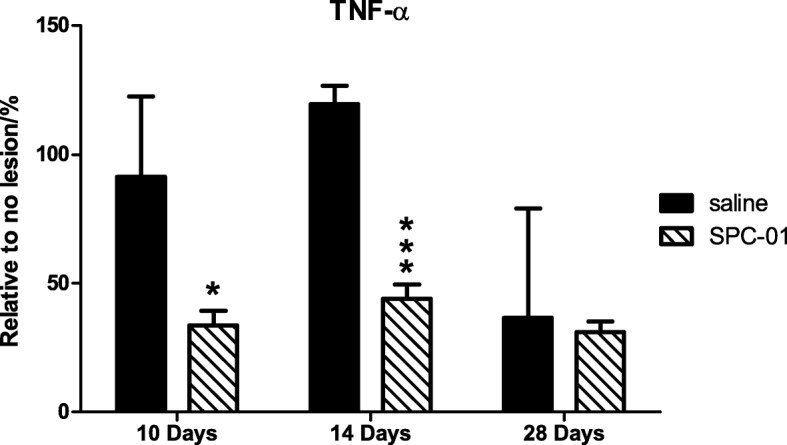
Levels of cytokine (TNF-α) were determined at 10, 14, and 28 days after SCI in animals receiving SPC-01 or saline. Transplantation of SPC-01 cells resulted in marked reduction of TNF-α levels 10 and 14 days after injury when compared with animals injected with saline only. Results are represented as a percentage relative to uninjured controls. Data are shown as mean and SEM, *p ≤ 0.05, ***p ≤ 0.001

Production of TNF-α cytokine was measured using the Multiplex technique at 10, 14, and 28 days after SCI with or without stem cell treatment. Increased levels of TNF-α were found after spinal cord injury with the highest measured 14 days after SCI. Treatment with SPC-01 cells resulted in strong inhibition of production of this pro-inflammatory cytokine 10 and 14 days after SCI (Fig. 4).

### Transplantation of SPC-01 cells results in tissue sparing

**Fig. 5:**
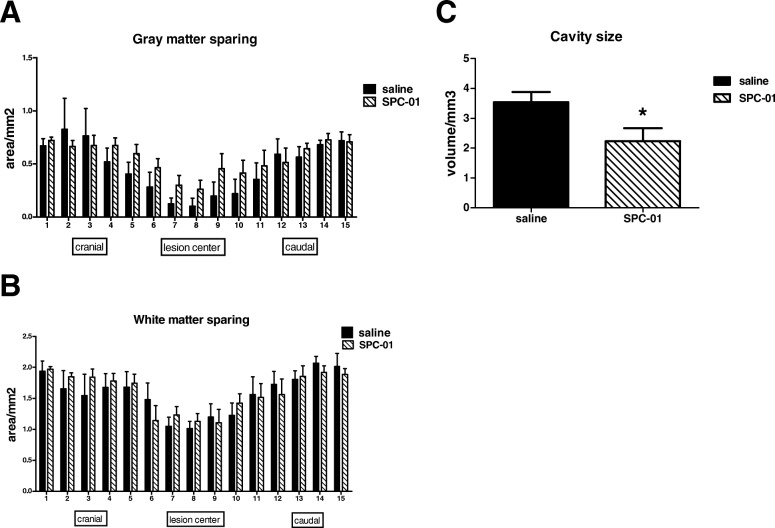
The spinal cord sections of rats at day 28 after SCI treated either with saline or SPC-01 were used to assess the gray (**a**) and white (**b**) matter tissue sparing. No local significant differences were found, but paired *t* test and two-way ANOVA revealed a significant difference in gray matter preservation between the treatment groups with *p* values of 0.0189 and 0.05, respectively (**a**). Rats transplanted with SPC-01 cells displayed a significantly smaller cavity size than rats treated with saline (**c**). Data are shown as mean and SEM, *p ≤ 0.05

Levels of gray and white matter sparing were assessed in the spinal sections of the treatment groups 28 days after SCI. No statistical difference was found in case of white matter sparing (Fig. 5b). Statistical analysis did not show significant local differences; however, paired *t* test revealed a significant difference between the treatment groups with rats implanted with SPC-01 cells displaying enhanced preservation of gray matter (*p* = 0.0189). Two-way ANOVA also revealed significant gray matter sparing in the SPC-01 group (*p* = 0.05) (Fig. 5a). In addition, the volume of cavities in the SPC-01 group was significantly smaller than in rats treated with saline only (Fig. 5c) despite comparable average lesion length in both groups.

### Astrogliosis is inhibited by SPC-01 cells

**Fig. 6:**
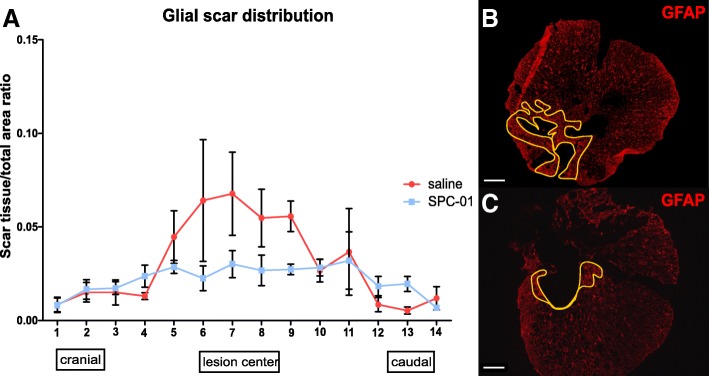
Glial scar distribution was assessed at day 28 after SCI using rat anti-GFAP-Cy3. Reduction in gliosis was observed in the central parts of the spinal cord lesion in rats transplanted with SPC-01 (**a**). Representative images of GFAP expression in saline-treated (**b**) or SPC-01 transplanted (**c**) rats with depicted glial scarring. Paired *t* test and two-way ANOVA revealed a strong significant difference between the treatment groups with *p* = 0.0068 and 0.0439, respectively. Data are shown as mean and SEM. Scale bars, 500 μm

The extent of the glial scar was determined 28 days after SCI in rats transplanted with SPC-01 cells or saline (Fig. 6). The spinal sections were stained against rat GFAP protein, a marker of host astrocyte activation. Our results show that transplantation of SPC-01 cells significantly inhibited the formation of glial scar and the activation of astrocytes. Statistical analysis did not show significant local differences, but paired *t* test demonstrated a strong significant difference between treatments (*p* = 0.0068), while two-way ANOVA also revealed the overall difference between the treatment groups (*p* = 0.0439).

## Discussion

We chose to transplant SPC-01 cells subacutely, 7 days after inducing SCI. Delayed transplantation of stem cells is an approach used by a number of investigators [26–31]. Cheng et al. observed improved results when neural stem cells were implanted 7 days after SCI [32]. A meta-analysis comparing acute and subacute cell transplantation revealed better results after subacute application [33]. Here, we show that the activity of p65 canonical NF-κB signaling pathway is significantly elevated after SCI when compared with untreated animals, which contributes to the transcription of pro-inflammatory and pro- and anti-apoptotic proteins leading to the natural promotion of healing [34]. As shown in Fig. 1a, b, we observed bimodal peaks of NF-κB p65 nuclear translocation in the course of the experiment. These peaks were detected at days 3 and 28 after inducing SCI. Our results suggest an upregulation in immune response on days 3 and 28 after SCI and further validate the strategy of delayed transplantation of stem cells at 7 days after SCI, when p65 NF-kB nuclear translocation is low. Previously, we observed functional improvements starting at day 28 after transplantation with SPC-01 cells. SPC-01 cells survived robustly and differentiated into all three neural lineages at the site of transplantation [21]. However, the transplanted cells matured at later stages (4 months after transplantation) as they remained rather immature 2 months after implantation. This implies that their observed therapeutic effect is not due to cell replacement but is likely of paracrine nature as suggested by increased gene expression of neural growth factor and *Nt3* in host tissue and in case of NGF in SPC-01 cells as well [23]. It has been shown that NT-3 reduces inflammation while enhancing regeneration in the injured spinal cords leading to functional improvements [35]. Our findings provided additional evidence to elucidate the mechanisms of neural progenitor cell therapy. Our results demonstrate a strong inhibitory effect of SPC-01 on TNF-α production and subsequently on NF-κB activation. We further observed that transplantation of SPC-01 cells into the spinal cord lesion resulted in a significant inhibition of nuclear translocation of p65 NF-kB on day 21, which we confirmed by means of immunohistochemistry and detection of levels of nuclear phosphorylated p65 NF-kB at Ser536 site (Figs. 2 and 3). Based on these findings, we can suggest that lower levels of p65 NF-kB translocation may partly be due to the inhibitory effect of SPC-01 cells on the levels of TNF-α, which was inhibited resulting in significantly lower production level when compared to the saline-treated rats. Decreased production of TNF-α at days 10 and 14 after SCI (3 and 7 days after transplantation) may have led to the inhibition of p65 NF-kB nuclear translocation as TNF-α is a known activator of canonical NF-κB pathway [7]. In addition, significantly lower numbers of p65 NF-kB expressing nuclei translocated in the gray matter in rats transplanted with SPC-01 cells may imply that lower number of glial cells contributed to the inflammatory reaction mediated by pro-inflammatory cytokines and chemokines. The inhibited immune response seems to be the major mechanism of the therapeutic effect of SPC-01 neural progenitor cells in recovery from SCI. Other studies using transplantation of neural progenitors in the treatment of SCI have provided evidence of reduced iNOS levels, cytokine production, and chemokine presence [17, 21, 22, 36], several of which are regulated by the NF-κB signaling pathway including IL-1β, IL-2, TNF-α, MIP1α, or RANTES. One of the implications and reasons of reduced NF-κB canonical pathway transcription activity and related changes in protein synthesis is lowering the load of infiltrating neutrophils, macrophages, and T lymphocytes. As demonstrated, chemokine MIP1α is known to recruit neutrophils and macrophages to the lesion sites as the first responders to injury where IL-1β contributes to macrophage activation [37]. Macrophages have been observed to enter the lesion site, and their numbers peak at days 3–4 after SCI [38], which corresponds with our finding of the first peak of NF-κB activity occurring at day 3 (Fig. 1a, b). Pro-inflammatory cytokines, such as IL-1β and TNF-α, released by present neutrophils, activated microglia, neurons, and astrocytes classically activate macrophages, which become the so-called M1 pro-inflammatory subtype [8, 39–41]. In fact, high levels of NF-κB activity along with STAT1, IRF5, and AP-1 are characteristic in this type of macrophages [42, 43]. Contrary to M1, the M2 macrophages are repair promoting, and their functions are regulated by several transcription factors, such as STAT6 and IRF4 [44]. Recruited macrophages are alternatively activated in the presence of IL-4 and IL-13 [45, 46]. Previous results from our group showed that transplantation of SPC-01 cells promotes the production of IL-4 at 14 and 28 days after SCI when compared with the saline-treated controls suggesting improved environment for generation of M2 macrophages [21] and possible alteration of the M1/M2 ratio with lower numbers of M1 macrophages. This would be complementary to our finding that SPC-01 cells reduce NF-κB activity 28 days after SCI while in saline controls, a second peak of NF-κB activity was observed, which was previously seen by Beck et al., where numbers of macrophages started increasing for the second time 28 days after SCI and peaked at 60 days after SCI [47]. Similarly, T-lymphocytes reactive to myelin basic protein (MBP) or myelin oligodendrocyte glycoprotein (MOG) are attracted to the lesion site by chemokines produced by microglia, neutrophils, macrophages, and astrocytes after approximately 1 week [48], where they differentiate into effector T cells in the presence of IL-2, indicating that peripheral tolerance by adaptive immunity has been overcome. Once in the spinal cord lesion, they further promote macrophage activity and secrete additional RANTES and MIP-1 chemokines attracting more macrophages from the periphery [49]. The numbers of T-lymphocytes have been shown to increase for at least 42 days [50] and persist for at least 70 days [49]. Previously, we reported that SPC-01 cells transplantation resulted in significantly lower production of IL-2 in all studied time points (10, 14, and 28 days after SCI) [21]. The second peak of NF-κB activation that we observed could be one represented by heightened macrophage activity also due to persistent T-lymphocyte infiltration. T lymphocytes work with antigen presenting cells (APCs), microglia, and macrophages, which in turn receive more signals to phagocytose and produce cytokines along with other reactive species [42]. Low levels of IL-2 after SPC-01 transplantation may reduce differentiation of T-lymphocytes and immune response reflected by low NF-κB activity. As previously shown, the neutrophil infiltration naturally subsides within a week after injury [51]. Contrary to that, macrophages persist in injured spinal cords for months. While their role in wound healing is essential, lower numbers through chronic stages and a milieu favoring their moderate activation can have a beneficial effect that can provide a more permissible environment for regeneration. In our study, we observed enhanced gray matter sparing with reduced volume of cavity size in rats treated with SPC-01 cells, which, in part, could be explained by the fact that the attenuated and modulated immune response along with production of neural growth factor by SPC-01 cells, may have resulted in improved tissue sparing and thus functional outcomes as shown in our previous study [21].

We suggest that transplantation of neural precursor cells resulted in inhibition of NF-κB activity and associated immune response not only in immune cells, resident or infiltrated, but also in astrocytes, which may have led to the reduction in astrocyte activation and formation of a smaller glial scar. It has been shown that NF-κB (p65/p50) is a major regulator of astrocyte activity after SCI [4]. Moreover, in vitro experiments have demonstrated that TNF-α induces NF-κB canonical pathway in astrocytes [4, 52]. Transgenic mice expressing an inhibitor of astroglial NF-κB showed improved motor function and reduced formation of glial scar along with better white matter preservation and smaller lesion size [4]. This would be in agreement with our hypothesis that reduced TNF-α at days 10 and 14 after SCI leads to the inhibition of NF-κB activity at day 28 after SCI. Moreover, Brambilla et al. not only demonstrated a lower production of astroglial chemokines but also significantly increased the production of IL-6 in the injured spinal cords of transgenic mice lacking active p65/p50 dimer. They concluded that IL-6 levels are dependent on production by astrocytes and that the canonical NF-κB pathway may be inhibitory in IL-6 astrocyte production [4]. Interestingly, transplantation of SPC-01 cells into injured spinal cords resulted in increased production of IL-6, which has been shown to promote axonal sprouting measured by GAP43 positivity [53] and corresponds with our findings [21]. Results of our study demonstrated that SPC-01 cells reduced the NF-kB p65 nuclear translocation, which resulted in smaller glial scar formation as well as increased production of IL-6 axonal regrowth enhancer.

## Conclusions

Here, we elucidated the activity pattern of canonical p65/p50 NF-κB signaling pathway in SCI and showed that it displays two peaks, 3 and 28 days after SCI. We further validated the approach of subacute cell transplantation 7 days after injury induction. We established that inhibition of NF-κB activity at day 28 after SCI in rats transplanted with neural progenitor cells SPC-01 reduced production of TNF-α. SPC-01 cells have strong immunomodulatory properties through inhibition of a major signaling pathway, which is based on our previous observation showing a reduction in levels of IL-2 involved in T-lymphocyte activation and increase in levels of IL-4, an important M2 inducer. It is conceivable that reduced NF-κB activity can be attributed to either inhibited M1 macrophages or their reduced numbers in the spinal cord as observed bimodal NF-κB activation correlates with peaks of macrophage and T-lymphocyte infiltration shown by other investigators [38, 47, 50]. Another possibility would be a shift in M1/M2 ratio in favor of M2, which are characterized by the activation of STAT6 and IRF4 transcription factors and not by NF-κB. We demonstrate here that treatment with SPC-01 cells leads to smaller glial scar formation, which could be due to the reduced astroglial p65/p50 NF-κB activity. This was found to be related to increased levels of IL-6, which promote the axonal sprouting, both of which were observed in our previous study after SPC-01 transplantation [21]. Moreover, the immunomodulatory effect of SPC-01 cells determined in our study could have resulted in enhanced gray matter sparing and reduced cavity size.

## Additional file

Additional file 1: SPC-01 graft. Transplanted cells were stained with human marker Ku80 to visualize the graft within spinal cord tissue. SPC-01 cells are indicated with white arrows (A). Grafts of SPC-01 cells formed dense clusters outlined in black, which were clearly distinguishable in both cresyl violet staining (B) and NFkB (p65) DAB staining (C). (PDF 755 kb)

## Abbreviations

ANOVA: Analysis of variance
AP-1: Activator protein 1
APCs: Antigen presenting cells
CNS: Central nervous system
DAMPs: Danger-associated molecular patterns
ELISA: Enzyme-linked immunosorbent assay
GAP43: Growth-associated protein 43
GFAP: Glial fibrillary acidic protein
IL: Interleukin
iNOS: Inducible nitric oxide synthase
IRF: Interferon regulatory factor
JNK: c-Jun N-terminal kinase
MBP: Myelin basic protein
MIP1α: Macrophage inflammatory protein 1α
MOG: Myelin oligodendrocyte glycoprotein
NF-κB: Nuclear factor kappa B
NGF: Nerve growth factor
NT-3: Neurotrophin-3
PAMPs: Pathogen-associated molecular patterns
RANTES: Regulated upon activation, normal T cell expressed, and secreted
SCI: Spinal cord injury
SPC-01: Spinal cord clonal line 1
STAT: Signal transducer and activator of transcription
TNF-α: Tumor necrosis factor alpha
