# Supplementary figures and images for: Transplantation of neural precursors generated from spinal progenitor cells reduces inflammation in spinal cord injury via NF-κB pathway inhibition
Source: J Neuroinflammation. 2019 Jan 17;16:12. doi: 10.1186/s12974-019-1394-7 (PMC6335809; doi:10.1186/s12974-019-1394-7)

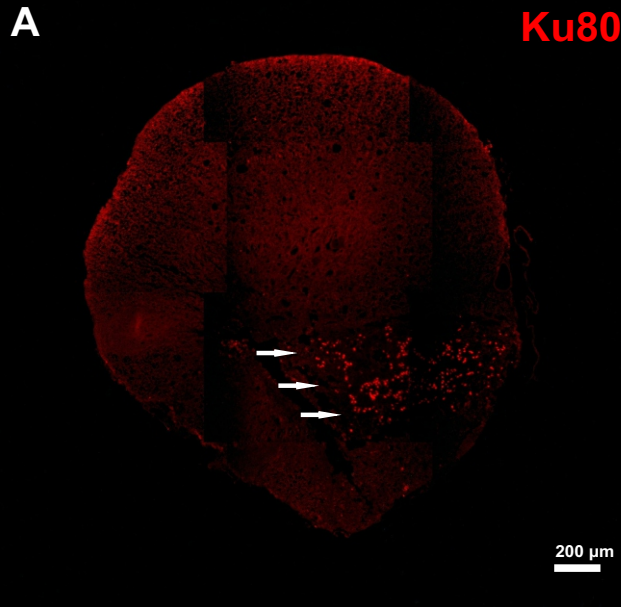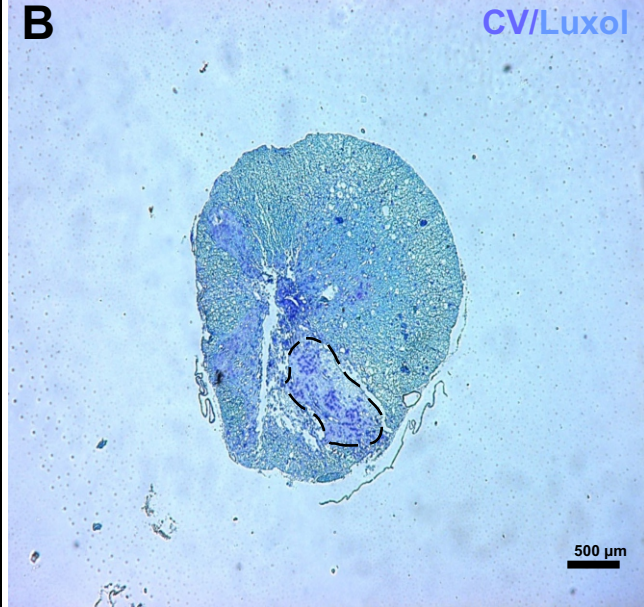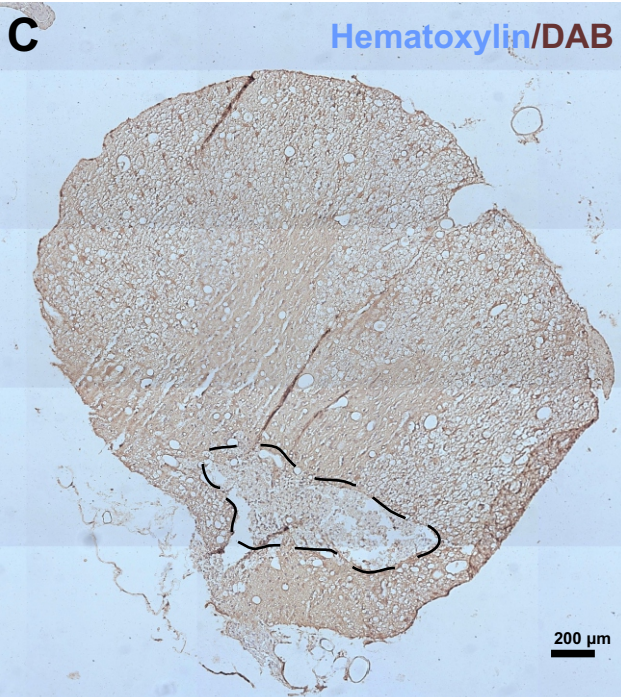

Supplement: Supplementary file 1 — SPC-01 graft. Transplanted cells were stained with human marker Ku80 to visualize the graft within spinal cord tissue. SPC-01 cells are indicated with white arrows (A). Grafts of SPC-01 cells formed dense clusters outlined in black, which were clearly distinguishable in both cresyl violet staining (B) and NFkB (p65) DAB staining (C). (PDF 755 kb) [file 12974_2019_1394_MOESM1_ESM.pdf]
